# Temporal and Spatial Scales Matter: Circannual Habitat Selection by Bird Communities in Vineyards

**DOI:** 10.1371/journal.pone.0170176

**Published:** 2017-02-01

**Authors:** Claire Guyot, Raphaël Arlettaz, Pius Korner, Alain Jacot

**Affiliations:** 1 Division of Conservation Biology, Institute of Ecology and Evolution, Department of Biology, University of Bern, Bern, Switzerland; 2 Field Station Valais, Swiss Ornithological Institute, Sion, Switzerland; 3 Swiss Ornithological Institute, Sempach, Switzerland; Universita degli Studi di Milano-Bicocca, ITALY

## Abstract

Vineyards are likely to be regionally important for wildlife, but we lack biodiversity studies in this agroecosystem which is undergoing a rapid management revolution. As vine cultivation is restricted to arid and warm climatic regions, biodiversity-friendly management would promote species typical of southern biomes. Vineyards are often intensively cultivated, mostly surrounded by few natural features and offering a fairly mineral appearance with little ground vegetation cover. Ground vegetation cover and composition may further strongly vary with respect to season, influencing patterns of habitat selection by ecological communities. We investigated season-specific bird-habitat associations to highlight the importance of semi-natural habitat features and vineyard ground vegetation cover throughout the year. Given that avian habitat selection varies according to taxa, guilds and spatial scale, we modelled bird-habitat associations in all months at two spatial scales using mixed effects regression models. At the landscape scale, birds were recorded along 10 1-km long transects in Southwestern Switzerland (February 2014 –January 2015). At the field scale, we compared the characteristics of visited and unvisited vineyard fields (hereafter called parcels). Bird abundance in vineyards tripled in winter compared to summer. Vineyards surrounded by a greater amount of hedges and small woods harboured higher bird abundance, species richness and diversity, especially during the winter season. Regarding ground vegetation, birds showed a season-specific habitat selection pattern, notably a marked preference for ground-vegetated parcels in winter and for intermediate vegetation cover in spring and summer. These season-specific preferences might be related to species-specific life histories: more insectivorous, ground-foraging species occur during the breeding season whereas granivores predominate in winter. These results highlight the importance of investigating habitat selection at different spatial scales and all along the annual cycle in order to draw practical, season-specific management recommendations for promoting avian biodiversity in farmland.

## Introduction

To counteract ongoing farmland biodiversity erosion, a wealth of evidence-based knowledge has been gathered during the last decades about wildlife in agroecosystems. Restoring farmland biodiversity requires information on the ecological requirements of different species at multiple spatial and temporal scales to capture the dynamics and resource needs of entire communities [1,2].

To complete their life cycle, birds have to fulfil diverse resource requirements varying between and within seasons. For instance, finding a suitable nesting place in close proximity to good foraging locations for provisioning food to chicks is a challenge commonly faced by parents during reproduction [1,3,4]. In winter, the main issue mostly concerns locating feeding patches offering sufficient food resources, with consequences for survival, especially late in the season when depletion of food supply may cause a “hungry gap”[5–8]. Not only inter-seasonal, but even intra-seasonal shifts in bird-habitat associations involving temporal changes in habitat suitability, food availability or diet have been demonstrated [9–15]. As an illustration, Douglas et al. [10] found that the use of field margins as foraging grounds by yellowhammers *Emberiza citrinella* markedly declined with the progress of the season, whilst use of cereal fields augmented, probably as a consequence of impeded food accessibility due to a growing ground vegetation within the field margins.

Since resources selection is operated in different habitat types and at different spatial scales to fulfil the crucial ecological requirements that may vary between species, the heterogeneity of the agricultural matrix must be considered at multiple spatial scales [16–18]. Increased habitat patchiness is thus known to offer a wider range of resources and to support higher biodiversity than monocultures [1,19]. **Landscape-scale heterogeneity** mainly affects birds through the proportions of different habitat types present in the overall matrix. Notably, the number of crop types and the relative amounts of woodland, steppe, wetlands strongly influence bird community composition and species richness [20]. Semi-natural boundary habitats such as field margins, hedges or forest edges are also well recognised to be valuable for birds in case of adequate management [1,21,22]. For example, optimal territories of red-backed shrikes *Lanius collurio* seem to harbour around 15–20% hedge cover among extensive mosaics of pasture and cultivation [23]. **Within fields**, sward structure constitutes a key factor for terrestrially-foraging and ground-breeding birds because it dictates both food availability (which is abundance modified by accessibility) and nesting opportunities [1,24–26]. A botanically and structurally (height and cover) diverse sward tends to harbour a higher food abundance and diversity [27] and makes seeds and invertebrates accessible to a broad range of bird species and foraging guilds [1]. Massive use of fertilizers has promoted uniformly higher and denser swards, which decreases food diversity, impedes foragers’ mobility, diminishes prey accessibility, and increases (actual or perceived) predation risk [28–30], with notorious deleterious effects on population growth rates [31]. Patches of bare ground among the ground vegetation cover have been associated with higher bird occupancy especially in winter (e.g. [32–35]), while an heterogeneous combination of patches of bare ground and vegetation cover appears to be key for terrestrially-feeding insectivorous birds during reproduction, providing support to the view that habitat selection results from a trade-off between food abundance and accessibility, which defines food availability [24,36,37]. Creating undrilled patches in the middle of cereal fields temporally prolongs breeding opportunities for skylarks *Alauda arvensis* probably because of enhanced food accessibility for foraging parents [38,39]. Hence, habitat selection is scale-dependent and will differ with respect to bird species, underpinning the importance of combining seasonal and spatial information for a better understanding of the actual constraints faced by bird communities occurring in vineyards.

The vegetation structure of vineyards also shows a great geographic and temporal variation with respect to management practice and environmental context. Vineyards can host rare and specialized plant and animal species, especially when they are managed extensively [40–44] and/or interspersed with natural elements such as hedgerows and surrounded by dry and warm natural habitat patches [42,45–49]. Viticulture has been shown to be regionally important for birds because it covers large areas, often on naturally biodiversity-rich, south-exposed xeric slopes. Nevertheless, there have been few attempts to study the habitat associations of integral vineyard bird communities on a year-round basis (but see [50,51]). In effect, research has been mostly autecological, furthermore focused on the breeding season [37,43,52]. Duarte et al. [53] demonstrated higher bird abundance, diversity and species richness in mechanically managed, vegetated vineyards compared to mineral and chemically managed viticulture areas, especially for insectivores. In line with these findings, the woodlark *Lullula arborea* has been shown to select patchy ground vegetation at the foraging site scale [37].

In this study, we focussed on bird communities occurring in vineyards submitted to a continental climate regime (large variation in conditions between seasons) within a major internal valley in Southwestern Switzerland. We investigated, at two spatial scales, the species-specific habitat associations of multiple bird species that use vineyards as breeding site, as migration stop-over site and as winter habitat. The main emphasis of this study was put on the season-specific effects of habitat characteristics on foraging activities. At the landscape scale, we studied which role marginal, (semi-)natural habitats play for birds within the agricultural matrix. At the field scale, we analysed the importance of ground vegetation structure and vineyard management for birds. By accounting for the broader landscape and assessing circannual variations in habitat selection patterns, our study aims at providing season-specific recommendations for bird-friendly vineyard management both outside and within the production area.

## Materials and Methods

### Study area & survey transects

The study was carried out in the Rhone river valley in the Canton of Valais (SW Switzerland, 540–780 m a.s.l.). This inner alpine, west-east oriented valley is characterized by a continental climate with little rain, hot summers and cold winters. The south-exposed slopes are dominated by vineyards covering roughly 50 km^2^ [37] whereas high-intensity fruit tree plantations represent the main agricultural activity on the plain [26]. Most vineyards in the study area are farmed following the integrated production (IP) protocol involving a reduction in insecticide and acaracide application. Since restriction in herbicide spraying is not mandatory for IP, about 95% of the vine fields still exhibit an almost exclusively mineral appearance due to systematic application of herbicides all over the ground [37]. Herbicide is applied to limit competition for water between vine plants and ground vegetation, especially under the dry circumstances of Central Valais [26]. The remaining 5% of the grape production area are still subjected to herbicide application, but only partially, which typically offers a mosaic with ca 50% ground vegetation cover alternating with bare ground patches. This is because most often every second plant row or inter-row is treated to combat ground vegetation. Organic vineyards, where vegetation is allowed to grow over the whole ground surface and where its removal can be performed only mechanically, make up scarcely 2% of the total area [37].

**Fig 1:**
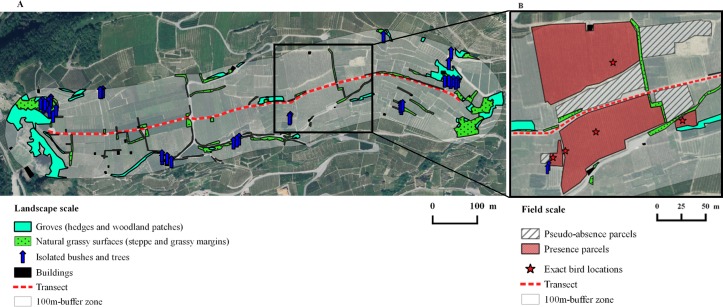
Study design. Satellite picture of a typical transect (St-Léonard as an example) surrounded by its arbitrarily defined buffer zone depicting study designs at the two scales considered. (A) Landscape scale with mapped habitat structures. (B) Field scale (B is an excerpt from A): parcels with bird observations depicted by stars (presence data) and parcels with absence of bird observations (pseudo-absence data). Reprinted from Swisstopo under a CC BY license, with permission from Alexandra Frank(see S1 File).

From February 2014 to January 2015, we surveyed 10 transects evenly distributed between Fully (46°08'43.0"N 7°07'30.5"E) and Leuk (46°19'03.5"N 7°37'59.7"E; see S1 Fig) [45]. The vineyards were selected to represent varying habitat characteristics. Four categories of landscape structures were mapped in August 2014 in a buffer zone of 100 m on both sides of each transect (Table 1A and Fig 1A): grove area, natural grassy area, number of isolated bushes and trees as well as number of buildings. Grove is here defined as a surface densely covered with bushes and trees, such as a hedge or a small patch consisting of woody plants. Natural grassy surfaces included surfaces covered with climatic steppe vegetation and grassy vineyard margins. Buildings mainly consisted of sheds among the vineyards. Landscape habitat structures mapped in the field were subsequently quantified into Quantum Geographic Information System version 2.6.1 (QGIS 2015 [54]). Landscape structures and exact start and end coordinates of transects can be found in S1 Table.

**Table 1:** Explanatory variables recorded for habitat selection modelling.

Variable category	Variable name	Variable type	Recording method	Definition
(A) LANDSCAPE SCALE				
**Landscape structure**	Grove cover	Continuous	QGIS	Proportion grove (% hedges and woodland patches) within the 100-m buffer zone around one transect.
	Natural grassy surface cover	Continuous	QGIS	Proportion grassy surfaces outside vine parcels (% steppe and vineyard margins) within the 100-m buffer zone around one transect.
	Isolated bushes & trees density	Continuous (discrete)	QGIS	Number of isolated bushes and trees within the 100-m buffer zone around one transect per km.
	Building density	Continuous (discrete)	QGIS	Number of buildings (mainly sheds) within the 100-m buffer zone around one transect per km.
(B) FIELD SCALE				
**Vegetation structure**	Green ground vegetation cover	Continuous	In the field	Visually estimated percentage of ground vegetation at the parcel scale (5%-precision, vines not considered).
	Brown ground vegetation cover	Continuous	In the field	Visually estimated percentage of ground vegetation at the parcel scale (5%-precision, vines not considered). Proxy for herbicide application and dry material.
	Ground vegetation height	Continuous	In the field	Visually estimated mean height of ground vegetation (cm).
**Vineyard management**	Vineyard cultivation mode	Categorical	In the field	Distance between plant rows: short for gobelets (typically ca.100-110 cm spacing), large for wires (ca. 120–200 cm spacing) [37].
	Grape vine abundance	Continuous (discrete)	In the field	Number of grape bunches counted on five vine plants. Every second vine plant located in a randomly selected row on the parcel was considered for quantifying grape abundance.
	Parcel area	Continuous	QGIS	Area of the parcel (m^2^).
**Distance to landscape structure**	Distance to nearest grove	Continuous	ArcGIS	Distance between a recorded bird observation or a random point and the border of the nearest grove in the same transect (m).
	Distance to nearest natural grassy surface	Continuous	ArcGIS	Distance between a recorded bird observation or a random point and the border of the nearest natural grassy surface in the same transect (m).
	Distance to nearest isolated bush or tree	Continuous	ArcGIS	Distance between a recorded bird observation or a random point and the nearest isolated bush or tree in the same transect (m).
	Distance to nearest building	Continuous	ArcGIS	Distance between a recorded bird observation or a random point and the nearest building border in the same transect (m).

(A) Variables considered at the landscape scale.

(B) Variables considered at the field scale. Vegetation structure and vineyard management variables were recorded both for presence and pseudo-absence parcels, whereas distances to landscape structures were compared between presence parcels and random points within the 100-m buffer zone around transects.

### Bird surveys

Bird surveys were performed using a line transect sampling method with a cut-off distance of 100 meters on each side (Fig 1A). For each of the 10 selected vineyards, one footpath of 1.12 ± 0.10 km (mean ± SE) following the topographic contour lines (whenever possible) was visited twice a month in a randomized order from February 2014 to January 2015, except in April when only 5 transects could be monitored during the second sampling session. During the breeding season (from April to mid-July), surveys only took place between sunrise and 11:00 [55]. In autumn and winter, sampling was performed between one hour after dawn and one hour before dusk in order to avoid biases caused by birds travelling between feeding and roosting sites [33]. Surveys only took place under non-adverse weather conditions (no precipitation, no or little wind). Bird counts and habitat characteristics of both actual observation locations (presence) and pseudo-absence locations (see later) were recorded using the application Biolovision v.0.21 (Biolovision SARL, Ardon) on a smartphone (Samsung Galaxy Note 3 SM-N9005, South Korea). Since transects were located on public roads or tracks, no permits were required for bird surveys.

#### Landscape scale surveys

The landscape scale comprised all birds and 4 landscape structures (Table 1A and Fig 1A) recorded within the area delimited by the 100-m buffer zone around transects, including birds flying relatively close to the ground (ca. ≤ 20 m). Observations of single birds or flocks of a given species were recorded as a single observation point per species. Bird abundance data was summed per species, survey and transect.

#### Field scale surveys

A parcel represents here a vine surface managed in a uniform way, which translates into homogeneous habitat characteristics within that field. It usually displays a different ground vegetation structure as well as a different cultivation mode (e.g. “gobelets” or wires, for a definition see [37]) from the neighbouring parcels, making parcel delimitation fairly obvious in the field. Whenever a bird was located in a specific vine parcel (meaning on the ground, on a vine plant or flushed from the parcel), the parcel was considered a “presence parcel”. If a bird flock was spread over more than one parcel, we recorded the number of parcels on which birds occurred (multiple presence parcels). We compared each presence parcel (used habitat) to one adjacent “pseudo-absence parcel” (available but unused habitat) [56] within the 100m-buffer, hence obtaining a paired design (Fig 1B). A pseudo-absence parcel had to be non-used by the same bird species during the same survey. For each observation at the field scale, vegetation structure and vineyard management variables were immediately quantified in the field, both in the presence and in the associated pseudo-absence parcel. Visually estimated ground vegetation structure variables were green cover, brown vegetation cover and mean vegetation height. Vineyard management variables included cultivation mode (gobelets or wires), grape abundance and parcel area. Grape abundance was considered as it may attract frugivores such as thrushes (Table 1B; [6,37]).

Given that the quality of a parcel as experienced by the birds might be conditional on semi-natural habitat features in the vicinity, we additionally measured the distance of the presence point locations (red stars in Fig 1B) and of random points to the closest landscape structures using GIS ESRI® ArcMap^TM^ 10.2.2 (ESRI Inc., Redlands, CA, USA [57]; Table 1B). More specifically, within each transect buffer zone, we generated as many random points as there were presence points in this buffer zone. Then, each random point was allocated to one presence point in a way to minimize the sum of the distances between random and presence points. The mean and standard deviation of the distances between paired random and presence points were 46.44 ± 32.45 m (range: 1.87–135.05 m). Random points were not forced to lie in the pseudo-absence parcels, because in such a design both random and presence points would mostly be very close to each other, making a potential effect of semi-natural structures very unlikely to be detected.

### Statistical analyses

#### Landscape scale

Landscape scale analyses included the four landscape structures and circular month variables (the cosinus and sinus of month values 1 to 12 scaled to the interval 0 to 2π) [58] in relation to bird abundances (survey transect counts of all species pooled or summing only the Fringillidae or the *Turdus* species separately), species richness and Shannon diversity (‘vegan’ R-package version 2.3–1. [59]). Since sample sizes of single species were usually insufficient to model species-specific habitat selection separately, some species commonly visiting vineyards were pooled into the genus *Turdus* (thrushes, 4 species: song thrush *Turdus philomelos*, mistle thrush *Turdus viscivorus*, fieldfare *Turdus pilaris* and blackbird *Turdus merula*) and the family of Fringillidae (finches, 6 species: chaffinch *Fringilla coelebs*, linnet *Carduelis cannabina*, goldfinch *Carduelis carduelis*, greenfinch *Carduelis chloris*, citril finch *Serinus citrinella* and serin *Serinus serinus*). Species richness refers to the total number of species encountered per transect, also beyond the above two pooled taxonomic groups. Concerning landscape explanatory variables, proportions were arcsin-square root transformed and counts (densities) log transformed. We arcsin-square-root transformed proportions and log-transformed counts or other variables that were strongly right-skewed to improve model fit and to better comply with model assumptions. If the log works better than the untransformed variable, this indicates that the (biological) effect is rather multiplicative than additive, and vice versa. The arcsin-square-root-transformation stretches small proportion values. A difference of 1% grove between two transects A and B has not the same effect for birds if A: 50% to B: 51% or if A: 1% to B: 2%. In the first case, it represents a small, in the second case a large change. Our transects exhibited a grove cover between 0 to 11%. We used arcsin-sqrt instead of logit due to the zeros in our sample. Values were then standardized (mean = 0, standard deviation = 1) to improve convergence of the model fitting algorithms. In case of (continuous) explanatory variables showing a Spearman correlation coefficient |r_s_| > 0.7, the biologically less meaningful variable was excluded from modelling. Because the number of potential models was large relative to our sample size due to various possible combinations of explanatory variables, the model selection procedure was conducted in two steps.

In a first step, overall habitat preferences (complete data set from the whole year) were investigated using Generalized Linear Mixed Models (GLMMs) from the ‘lme4’ R-package (version 1.1–10, [60]) with ‘transect’ and ‘date’ set as random effects to account for repeated visits per transect and per day. An observation level random factor was added to correct for overdispersion when necessary [61,62]. Except for species richness and diversity, the log of the transect length was used as an offset [63]. The potential influence of transect area on species richness was evaluated by including the linear, quadratic and cubic effects of the area in the models. Polynomial and linear terms were successively discarded when they met the following two criteria: their 95%-credible intervals included zero and the effect size of the squared effect was smaller than the one of the linear effect. In all cases, we ended up with species richness models without any area variables (note that the area of transects did not vary strongly). Time since sunrise (time as the transect visit started relative to the time between sunrise and sunset) was included as a covariate to correct for known variability in bird activity according to daytime [64]. For abundance data and species richness, a Poisson model with a log-link function was used. For species diversity, a Gaussian error structure was assumed. In all full models, random slopes of the predictors were investigated but never improved the model fit according to the AICc (ΔAICc > 20). Also, the additional variance explained by the model when random slopes were included was always near zero and maximally 0.2% (calculated by Δdeviance/null deviance). On the other hand, excluding transect as random intercept produced a much larger AICc value (Δ4.7 for the Shannon Index and > 40 for the other outcome variables), hence transect was always retained as a random effect.

To generate the set of candidate models (differing only in the fixed effects), all possible combinations of the four landscape structure variables (Table 1A and Fig 1A) were fitted using the ‘dredge’ function of the ‘MuMIn’ R-package (version 1.15.6., [65]). Model selection was based on Akaike’s Information Criterion corrected for small sample size (AICc; [66,67]). Semi-variograms did not show substantial spatial autocorrelation in the data ('gstat' R-package version 1.1–3, [68]).

In a second step, each of the landscape variables figuring in the best model (with the lowest AICc; but always including the relative time since sunrise) of overall habitat preferences was used separately in combination with circular month variables to further investigate seasonal variation in habitat selection patterns [10]. For this purpose, a set of candidate models including all possible combinations of the habitat variable and the cosinus and sinus of month (values 1 to 12 scaled to the interval 0 to 2π; hereafter “cosmonth” and “sinmonth”) were, again, fitted by means of the ‘dredge’ function and ranked according to AICc (possible combinations of predictors included models with interactions except for the interaction between cosmonth and sinmonth). A significant main effect of cosmonth can be interpreted as a difference in bird abundance between winter and summer (a positive coefficient estimate relates to a higher abundance in winter, a negative coefficient to a higher abundance in summer). Sinmonth analogously refers to increased numbers in spring (positive estimate) vs decreased numbers in autumn (negative estimate). A significant interaction between a habitat variable and cosmonth and sinmonth suggests a variation of the birds’ affinity for that habitat variable across seasons. Model assumptions were checked using residual plots (including autocorrelation among the residuals), overdispersion was assessed using the ‘dispersion_glmer’ function from the ‘blmeco’ R-package (version 1.1, [63]). Semi-variograms did not show substantial spatial autocorrelation in the data ('gstat' R-package version 1.1–3, [68]).

#### Field scale

In field scale analyses, bird occurrence (presence = 1 vs. pseudo-absence = 0 for all species pooled as well as Fringillidae and *Turdus* species considered separately) was analysed in relation to the vegetation structure, vineyard management, distances to landscape elements and circular month variables. All explanatory variables were standardized to facilitate model fitting and comparison of effect sizes while checking for collinearity (as above, only the biologically most meaningful of two variables was kept when Spearman |r_s_| > 0.7). The same two habitat selection steps as described for the landscape scale were done, but preceded by an additional step due to the larger number of predictors at the field scale compared to the landscape scale; hence the following three steps were conducted:

First, in order to reduce the number of explanatory variables we pre-selected habitat variables. Vineyard management variables and distances to landscape structures showing a trend (*P* ≤ 0.1) in univariate models were used in the second modelling step. The vegetation structure variables were each tested for the presence of an optimum by including their linear and quadratic effects in a single model, because of a likely trade-off between food abundance (more food in denser vegetation) and accessibility (reduced accessibility in denser vegetation, hence an intermediate vegetation density may be optimal for the birds) [24]. Quadratic terms were discarded when they met the following two criteria: their 95%-credible intervals included zero and the effect size of the squared effect was smaller than the one of the linear effect. This procedure ensured that no squared effect potentially playing a major role in habitat selection was missed. Linear effects of vegetation structure variables were always included because of their particular interest in this study. These pre-selected variables were then used for the second step. While transect as random effect was suggested to be important at the landscape scale (see above), AICc values and the amount of explained variance suggested to exclude random effects at the field scale. Some random slope models explained up to 6.7% more variance, but mostly the additional variance explained was < 2% and the AICc was always lowest in the model without random effects (ΔAICc > 2).

In the second step (corresponding to the first step at the landscape scale), overall habitat preferences were modelled by means of hierarchical logistic regression models (logit-link function) with the random effects ‘transect’ and ‘paired ID’ (identifying pairs of presence and pseudo-absence parcels (for vegetation and management variables) and presence and random points (for distance to landscape structures)). As in the first step of the analysis at the field scale, random effects were not suggested to be important according to AICc and amount of variance explained, neither in the full models, nor in the selected best models (see below). The set of candidate models was created by combining all vegetation structure variables (linear and squared effects retained from the first step) and other variables showing a trend in univariate models in all possible combinations; these models were then ranked according to AICc (using the function ‘dredge’ as for the landscape scale).

Third (corresponding to the second step at the landscape scale), habitat variables occurring in the most parsimonious model (lowest AICc) were investigated for seasonal habitat selection patterns as explained for the landscape scale analyses above. Note that each habitat variable (the linear and, potentially, the quadratic term) was tested individually; e.g. for the pooled species, the most parsimonious model included the predictors green, green^2^ and brown, hence we created two models to test for seasonal effects (one containing green and green^2^, the other including brown). This approach of partial analyses was adopted because at this stage we were specifically interested in the question whether important habitat variables showed some seasonality in their effect on the outcome variable; including several habitat variables would have led to too many candidate models.

#### Model averaging and predictions

Finally, at both spatial scales, we used model averaging over the set of competitive models (ΔAICc ≤ 2; [66]) to estimate coefficients, SE, and p-values for each habitat predictor with the ‘full average’ output from the ‘model.avg’ function (‘MuMIn’ R-package version 1.15.6., [65]). Fixed effects always present in the competitive models and/or whose model-averaged p-values were significant were considered to probably influence habitat selection by birds. To get model-averaged predictions (mainly to produce effect plots), we used the Bayesian framework. We drew samples from the joint posterior distributions using the function ‘sim’ (‘arm’ R-package version 1.8–6., [69]). The number of samples per joint posterior (i.e. per model, since there is one joint posterior per model) was proportional to the model weights as suggested by Burnham & Anderson 2002. This procedure returns model-averaged posterior distributions for fitted values, which were used to draw effect plots (the mean of the posterior was used as the best fitting line and a 95% interval to depict the uncertainty of the prediction) [63].

All statistical analyses were conducted in R version 3.1.3 [70]. The response variable at the field scale (called predicted occurrence probability) should be interpreted as the probability of selecting a habitat relative to the other available habitats in our study area [56].

## Results

In total, we recorded 8719 individuals from 4421 observations (excluding pseudo-absences) belonging to 66 bird species within the 100-m buffer of all transects. At the field scale, 886 individuals from 298 presence parcels totalling 29 species were recorded (see S2 Table for species list), together with 291 corresponding pseudo-absence parcels and random points (in a very few cases, the same pseudo-absence parcel was paired to more than one surrounding presence parcel during the same survey).

### Seasonal bird abundances

**Fig 2:**
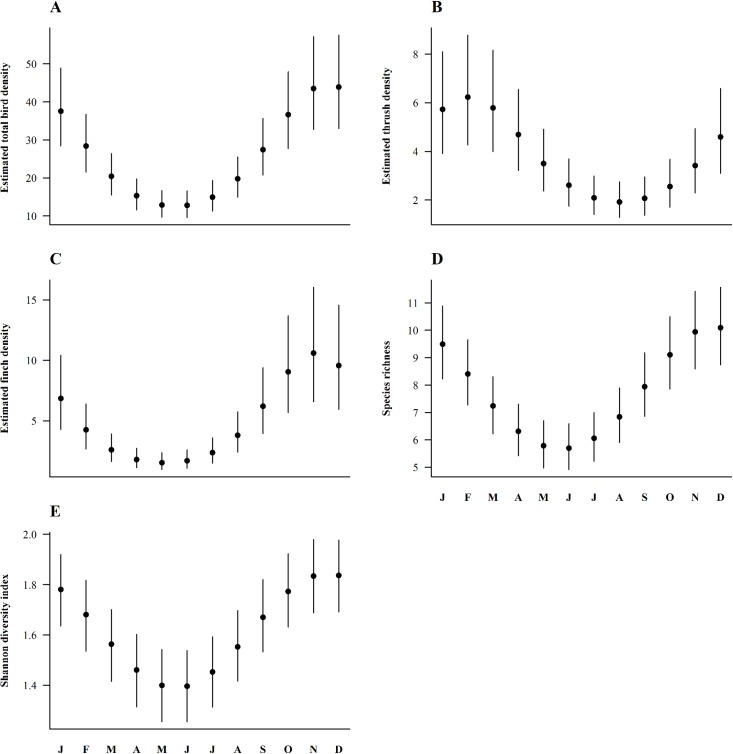
Model-averaged predictions of seasonal bird abundance at the landscape scale. (A) Pooled species. (B) *Turdus* spp. (thrushes). (C) Fringillidae (finches). (D) Species richness. (E) Shannon diversity. These relationships demonstrate a significant (model-averaged) effect of cosmonth and/or sinmonth on habitat selection (see Table 3B). Shown are model-averaged bird density predictions (per 20 ha) from Poisson regression models with 95%-Bayesian credible intervals. The letters on the x-axis stand for the first letter of each month from J: January to D: December.

A significant effect of cosmonth (depicting a contrast between summer and winter bird abundance) was noted in all competitive models for the abundance of pooled species and finches, species richness as well as for Shannon diversity. Sinmonth (contrast between spring and autumn abundance) also significantly affected pooled species, thrushes and finches abundances (Table 2B). For species richness and Shannon diversity, sinmonth (mostly) only showed a trend (Table 2B). For all response variables except thrush abundance, cosmonth had a stronger effect (larger effect size) than sinmonth (Table 3B). Predicted overall bird abundance (pooled species) was more than three times higher in winter (December) compared to summer according to the model-averaged parameter estimates (June; Table 3B and Fig 2A). Thrushes occurred in about threefold larger numbers in late winter compared to summer, with maximal and minimal predicted densities in February and August, respectively (Table 3B and Fig 2B). Finches’ density also significantly differed between all four seasons (Table 3B), peaking in November (ca. 11 individuals per 20 ha) and then decreasing until May (ca. 2 individuals per 20 ha; Fig 2C). Species richness was greater in winter (on average 10 species in December) than in summer (6 species in June on average; Table 3B and Fig 2D). Finally, Shannon diversity was also higher in winter (average index of 1.85 in December) compared to summer (average index of 1.40 in June; Table 3B and Fig 2E).

**Table 2:** Competitive models from the overall and seasonal model selection procedures at both spatial scales.

Response variable	# Candidate models	Competitive models (ΔAICc ≤ 2)	Df	Deviance	ΔAICc	Weight	Sample size (# Obs./Transects)
(A) LANDSCAPE SCALE: OVERALL HABITAT SELECTION							
**Pooled species abundance**	16	**G** + **t**	6	2090.26	0.00	0.254	235/10
		**T**	5	2093.73	1.37	0.128	
		**G** + IBT + **t**	7	2089.91	1.78	0.104	
		*G* + NGS + **t**	7	2090.12	1.99	0.094	
**Thrush abundance**	16	**G** + *NGS* + **t**	7	1306.31	0.00	0.203	235/10
		**G** + **t**	6	1308.80	0.37	0.169	
		**T**	5	1311.90	1.36	0.103	
		**G** + B + NGS + **t**	8	1305.94	1.77	0.084	
		**G** + IBT + **t**	7	1308.19	1.88	0.079	
		**G** + B + **t**	7	1308.29	1.97	0.076	
**Finch abundance**	16	T	5	1553.76	0.00	0.214	235/10
		G + t	6	1553.09	1.44	0.105	
		NGS + t	6	1553.26	1.60	0.096	
		B + t	6	1553.52	1.86	0.085	
		IBT + t	6	1553.54	1.88	0.084	
**Species richness**	16	**G** + **t**	5	1229.75	0.00	0.286	235/10
		**G** + IBT + **t**	6	1228.70	1.06	0.168	
		**G** + NGS + **t**	6	1229.51	1.87	0.112	
**Shannon diversity**	16	**G** + IBT + **t**	7	305.92	0.00	0.215	235/10
		*G* + **t**	6	308.16	0.11	0.203	
		**G** + IBT + B + **t**	8	305.49	1.72	0.091	
		**G** + NGS + **t**	7	307.72	1.80	0.087	
(B) LANDSCAPE SCALE: SEASONAL HABITAT SELECTION							
**Pooled species abundance**	13	*G* + **cosmonth** + **sinmonth** + **G:cosmonth** + **t**	9	2032.58	0.00	0.542	235/10
**Thrush abundance**	13	*G* + **cosmonth** + **sinmonth** + *G*:*cosmonth* + **t**	9	1284.93	0.00	0.279	235/10
		*G* + **cosmonth** + **sinmonth** + **t**	8	1287.61	0.52	0.215	
		**G** + **cosmonth** + **sinmonth** + **G:cosmonth** + G:sinmonth + **t**	10	1283.78	1.04	0.166	
		**cosmonth** + **sinmonth** + **t**	7	1290.83	1.60	0.125	
		**G** + **cosmonth** + **sinmonth** + G:sinmonth + **t**	9	1286.57	1.65	0.123	
**Thrush abundance**	13	**cosmonth** + **sinmonth** + **t**	7	1290.83	0.00	0.504	235/10
**Finch abundance**	4	**cosmonth** + **sinmonth** + **t**	7	1519.58	0.00	0.985	235/10
**Species richness**	13	**G** + **cosmonth** + *sinmonth* + *G*:*cosmonth* + **t**	8	1184.95	0.00	0.314	235/10
		**G** + **cosmonth** + *G*:*cosmonth* + **t**	7	1188.02	0.92	0.198	
		**G** + **cosmonth** + *sinmonth* + **t**	7	1188.3	1.21	0.172	
**Shannon diversity**	13	*G* + **cosmonth** + *sinmonth* + **G:cosmonth** + **t**	9	279.89	0.00	0.465	235/10
		*G* + **cosmonth** + **G:cosmonth** + **t**	8	282.91	0.86	0.303	
		*G* + **cosmonth** + *sinmonth* + **G:cosmonth** + G:sinmonth + **t**	10	279.54	1.83	0.186	
**Shannon diversity**	13	**cosmonth** + *sinmonth* + **t**	7	292.64	0.00	0.382	235/10
		**cosmonth** + **t**	6	295.83	1.06	0.225	
(C) FIELD SCALE: OVERALL HABITAT SELECTION							
**Pooled species occurrence**	12	**green + green^2 + brown**	4	754.51	0.00	0.501	589
		**green + green^2** + *brown* + vegheight	5	754.18	1.70	0.214	
		**green + green^2**	3	758.46	1.93	0.191	
**Thrush occurrence**	16	**green + dist_NGS**	3	261.52	0.00	0.351	197
		**green** + vegheight + **dist_NGS**	4	260.61	1.18	0.195	
		**green** + brown + **dist_NGS**	4	261.41	1.98	0.131	
**Finch occurrence**	12	**green + green^2 + brown**	4	193.91	0.00	0.392	165
		**green + green^2 +** *brown* **+** vegheight	5	192.05	0.26	0.344	
(D) FIELD SCALE: SEASONAL HABITAT SELECTION							
**Pooled species occurrence**	38	**green + green^2** + cosmonth + sinmonth + **green:cosmonth + green:sinmonth**	7	729.40	0.00	0.397	589
		**green + green^2** + *cosmonth* + sinmonth + **green:cosmonth** + green^2:cosmonth + **green:sinmonth**	8	728.40	1.05	0.234	
		**green + green^2** + cosmonth + sinmonth + **green:cosmonth + green:sinmonth** + green^2:sinmonth	8	728.77	1.43	0.195	
**Pooled species occurrence**	13	**brown** + cosmonth + **brown:cosmonth**	4	801.29	0.00	0.382	589
		**brown** + cosmonth + sinmonth + **brown:cosmonth** + *brown*:*sinmonth*	6	798.09	0.88	0.246	
		**brown** + cosmonth + sinmonth + **brown:cosmonth**	5	800.90	1.65	0.168	
**Thrush occurrence**	13	**green** + cosmonth + sinmonth + **green:cosmonth + green:sinmonth**	6	234.21	0.00	0.893	197
**Thrush occurrence**	13	**dist_NGS**	2	267.29	0.00	0.373	197
		**dist_NGS** + cosmonth	3	267.12	1.90	0.144	
**Finch occurrence**	38	**green + green^2** + cosmonth + green:cosmonth + *green^2*:*cosmonth*	6	191.75	0.00	0.190	165
		**green + green^2** + cosmonth + *green^2*:*cosmonth*	5	194.75	0.84	0.125	
		**green + green^2**	3	199.12	0.99	0.116	
		**green + green^2** + cosmonth	4	197.71	1.67	0.082	
		**green + green^2** + cosmonth + green:cosmonth	5	195.66	1.75	0.079	
**Finch occurrence**	13	**Brown**	2	218.81	0.00	0.346	165
		**brown** + sinmonth	3	217.80	1.06	0.203	
		**brown** + cosmonth	3	218.70	1.97	0.130	

(A) Overall habitat selection at the landscape scale. (B) Seasonal habitat selection at the landscape scale. (C) Overall habitat selection at the field scale. (D) Seasonal habitat selection at the field scale. At the landscape scale, Poisson GLMMs were fitted for abundance data, whereas Normal LMM was used for Shannon diversity. *t* stands for the systematically included covariate “relative time since sunrise”. At the field scale, binomial GLMs were applied to presence/pseudo-absence data. The number of candidate models was 16 for the overall habitat selection at the landscape scale (all combinations of four predictor variables), but depended on the number of variables pre-selected for the models at the field scale (see Material and Methods). Seasonal models were conducted for each habitat variable retained in the most parsimonious overall habitat model; these models contained only one or two variables at the landscape scale and always two at the field scale (hence there are always two seasonal models per outcome variable at the field scale). Explanatory variables are written in bold when significant (*P* ≤ 0.05) and in italics when showing a trend (*P* ≤ 0.1). G: Grove cover (% hedges and woodland patches); IBT: Isolated bushes and trees density; NGA: Natural grassy surface cover (% steppe and grassy margins); B: Building density; green: Green ground vegetation cover; brown: Brown ground vegetation cover; dist_NGS: Distance to nearest natural grassy surface; vegheight: Ground vegetation height.

**Table 3:** Model-averaged parameter estimates.

Explanatory variable	Pooled species			*Turdus* species (thrushes)			Fringillidae species (finches)			Species richness			Shannon diversity		
	Estimate	SE	P-value	Estimate	SE	P-value	Estimate	SE	P-value	Estimate	SE	P-value	Estimate	SE	P-value
(A) LANDSCAPE SCALE: OVERALL HABITAT SELECTION															
**Intercept**	3.12	0.12	<0.001***	1.23	0.15	<0.001***	1.33	0.18	<0.001***	2.01	0.06	<0.001***	1.61	0.05	<0.001***
**Grove cover**	0.19	0.15	0.203	0.28	0.19	0.129	0.02	0.09	0.780	0.17	0.07	0.008**	0.13	0.05	0.016*
**Natural grassy surface cover**	-0.01	0.05	0.887	-0.10	0.16	0.526	0.02	0.08	0.808	-0.01	0.03	0.841	-0.01	0.02	0.828
**Isolated bushes and trees density (per 20 ha)**	-0.01	0.06	0.826	-0.01	0.06	0.834	-0.01	0.07	0.869	-0.02	0.05	0.670	-0.04	0.06	0.446
**Building density (per 20 ha)**	-	-	-	0.00	0.09	0.981	-0.01	0.07	0.864	-	-	-	0.00	0.02	0.827
**Relative time since sunrise**	-0.25	0.05	<0.001***	-0.48	0.07	<0.001***	-0.13	0.10	0.177	-0.17	0.03	<0.001***	-0.22	0.03	<0.001***
(B) LANDSCAPE SCALE: SEASONAL HABITAT SELECTION															
**Intercept**	3.16	0.12	<0.001***	1.22	0.15	<0.001***	1.37	0.18	<0.001***	2.02	0.06	<0.001***	1.62	0.05	<0.001***
**Grove cover**	0.22	0.11	0.060.	0.23	0.16	0.146	NA	NA	NA	0.16	0.06	0.011*	0.11	0.05	0.031*
**Cosmonth**	0.62	0.08	<0.001***	0.29	0.12	0.020*	0.86	0.15	<0.001***	0.29	0.04	<0.001***	0.22	0.05	<0.001***
**Sinmonth**	-0.15	0.07	0.038*	0.52	0.12	<0.001***	-0.43	0.13	<0.001***	-0.05	0.04	0.276	-0.05	0.05	0.303
**Grove:cosmonth**	0.17	0.06	0.005**	0.07	0.10	0.453	NA	NA	NA	0.05	0.04	0.243	0.12	0.04	0.002**
**Grove:sinmonth**	-	-	-	-0.03	0.07	0.654	NA	NA	NA	-	-	-	0.00	0.02	0.819
**Relative time since sunrise**	-0.40	0.05	<0.001***	-0.52	0.07	<0.001***	-0.40	0.10	<0.001***	-0.26	0.03	<0.001***	-0.29	0.03	<0.001***
**Intercept**				1.22	0.17	<0.001***									
**Natural grassy surface cover**				NA	NA	NA									
**Cosmonth**				0.29	0.12	0.016*									
**Sinmonth**				0.51	0.12	<0.001***									
**NGS:cosmonth**				-	-	-									
**NGS:sinmonth**				-	-	-									
**Relative time since sunrise**				-0.52	0.07	<0.001***									
**Intercept**													1.62	0.06	<0.001***
**Isolated bushes and trees density (per 20 ha)**													-	-	-
**Cosmonth**													0.22	0.05	<0.001***
**Sinmonth**													-0.05	0.05	0.336
**IBT:cosmonth**													-	-	-
**IBT:sinmonth**													-	-	-
**Relative time since sunrise**													-0.29	0.03	<0.001***
(C) FIELD SCALE: OVERALL HABITAT SELECTION															
**Intercept**	0.03	0.09	0.696	0.06	0.15	0.703	0.04	0.18	0.812						
**Green cover**	0.55	0.09	<0.001 ***	0.38	0.16	0.021 *	0.68	0.19	<0.001 ***						
**Green cover**^**2**^	-0.31	0.09	<0.001 ***	NA	NA	NA	-0.47	0.19	0.012*						
**Brown cover**	0.14	0.11	0.196	-0.01	0.07	0.891	0.43	0.21	0.048 *						
**Vegetation height**	0.01	0.05	0.804	-0.04	0.11	0.692	0.13	0.20	0.520						
**Distance to nearest natural grassy surface**	NA	NA	NA	-0.35	0.15	0.022 *	NA	NA	NA						
(D) FIELD SCALE: SEASONAL HABITAT SELECTION															
**Intercept**	0.10	0.09	0.317	0.27	0.23	0.232	0.00	0.19	0.990						
**Green cover**	0.64	0.11	<0.001 ***	0.84	0.33	0.012 *	0.81	0.21	<0.001 ***						
**Green cover**^**2**^	-0.33	0.10	0.001 **	NA	NA	NA	-0.59	0.19	0.002 **						
**Cosmonth**	0.23	0.14	0.099.	0.47	0.33	0.156	0.32	0.30	0.294						
**Sinmonth**	0.05	0.13	0.721	0.28	0.24	0.250	-	-	-						
**Green : cosmonth**	0.73	0.19	<0.001 ***	1.75	0.49	<0.001 ***	0.26	0.40	0.509						
**Green : sinmonth**	0.41	0.13	0.002 **	0.87	0.32	0.007 **	-	-	-						
**Green**^**2**^** : cosmonth**	0.05	0.12	0.687	NA	NA	NA	0.30	0.38	0.419						
**Green**^**2**^**: sinmonth**	0.02	0.08	0.753	NA	NA	NA	-	-	-						
**Intercept**	0.06	0.09	0.515				0.05	0.16	0.761						
**Brown cover**	0.35	0.12	0.006 **				0.63	0.24	0.008 **						
**Cosmonth**	0.02	0.12	0.843				-0.01	0.11	0.891						
**Sinmonth**	-0.06	0.11	0.587				-0.07	0.18	0.676						
**Brown : cosmonth**	0.38	0.15	0.010 **				-	-	-						
**Brown : sinmonth**	-0.08	0.16	0.589				-	-	-						
**Intercept**				0.06	0.15	0.710									
**Distance to nearest grassy natural surface**				-0.35	0.15	0.020 *									
**Cosmonth**				0.03	0.13	0.841									
**Sinmonth**				-	-	-									
**Distance to nearest natural grassy surface: cosmonth**				-	-	-									
**Distance to nearest natural grassy surface: sinmonth**				-	-	-									

Model-averaged parameter estimates, standard errors (SE) and P-values from the ‘model-avg’ function [65] were considered for variables occurring in the respective sets of competitive models for (A) Overall habitat selection at the landscape scale. (B) Seasonal habitat selection at the landscape. (C) Overall habitat selection at the field scale. (D) Seasonal habitat selection at the field scale. Model averaging was performed over the set of competitive models (ΔAICc ≤ 2; see Table 2).

NAs stand for explanatory variables which were not tested for the corresponding response variables, whereas ‘-‘ means that the explanatory variable was tested but not retained in the competitive models for that response variable. Significance

*P < 0*.*1*.

*P < 0.05.

**P < 0.01.

***P < 0.001.

### Landscape scale habitat selection

**Fig 3:**
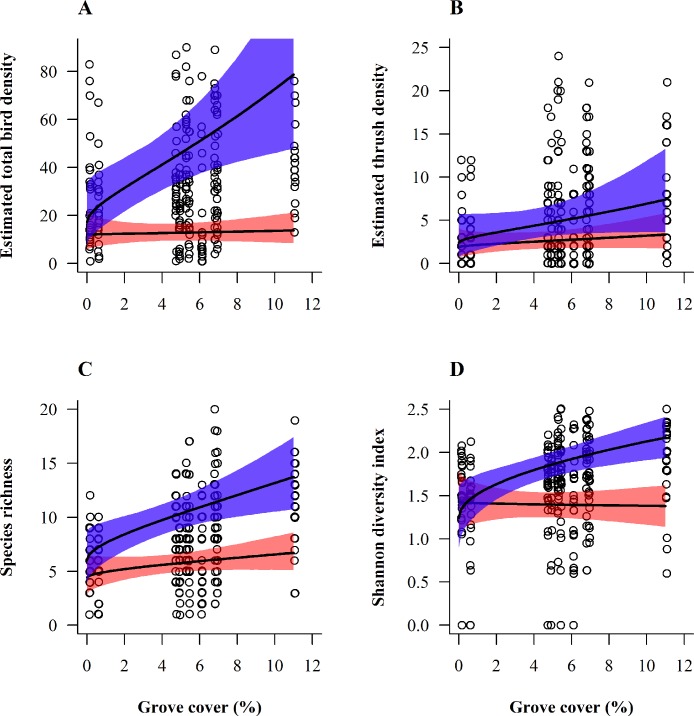
Model-averaged, seasonal relationships between grove cover and bird density at the landscape scale. (A) Pooled species. (B) *Turdus* spp. (thrushes). (C) Species richness. (D) Shannon diversity index. The habitat preference for greater grove cover was dependent of season. The two months “June” and “December” were retained for plotting predictions because they reflect the greatest changes in habitat selection between summer and winter (extremes). Shown are model-averaged bird density predictions (per 20 ha) from Poisson regression models (overall habitat selection) with 95%-Bayesian credible intervals (delimited by grey areas). Predicted estimates were allowed to vary with the habitat variable under consideration, while other explanatory variables present in the average model were held constant at their mean values. Circles represent raw data of the entire year.

For pooled species and thrush abundances, species richness and Shannon diversity, grove cover (hedges and woody patches) was the only significant landscape variable occurring in most competitive models (Table 2A). In all four cases, the effect of grove cover on birds varied according to season (Table 2B and Fig 3). However, notice that after model averaging, the interaction between grove and cosmonth only remained significant for pooled species abundance and Shannon diversity (Table 3B). Predictions showed a positive effect of increased grove proportion among vineyards for overall bird abundance, thrush abundance, species richness and diversity, with an even stronger positive effect in winter (Fig 3). In spite of the very low proportion of groves (mean ± SE: 5.21 ± 3.12% cover per transect), total number of individuals was predicted to at least quadruple in winter (December) whereas it would only increase by a factor of ca. 1.2 in summer (June) with its increase from 0 to 11% (Fig 3A). Similarly, species richness would increase by a factor of ca. 2.3 in winter against 1.5 in summer with such a grove proportion enhancement (Fig 3C). On the contrary, density of isolated bushes and trees and buildings as well as proportion of natural grassy areas did not have a significant effect (Table 3A). Furthermore, we did not detect any strong preference of finches for any of the considered landscape variables (“null model” including only the time covariate as best model in Table 2A; no significant model-averaged parameter estimate in Table 3A).

### Field scale habitat selection

**Fig 4:**
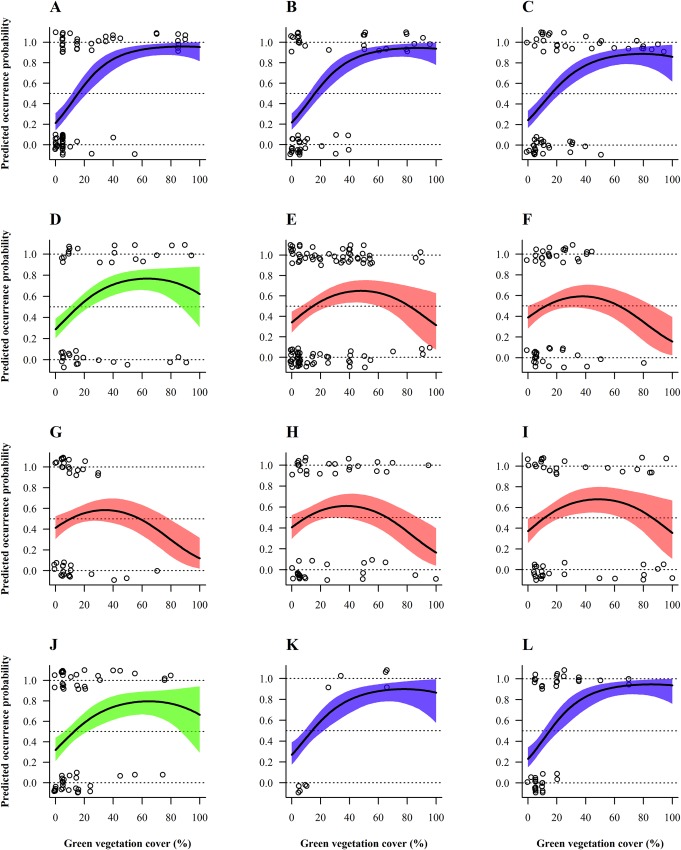
Model-averaged predicted seasonal bird occurrence probability depending on green ground vegetation cover and month. From (A) January to (L) December. Selection for green ground vegetation cover by pooled species significantly varied between seasons (months) at the field scale. 95%- Bayesian credible intervals are depicted by different coloured belts representing contrasting selection patterns. Circles represent raw data of the entire year. Occurrence probabilities greater than 0.5 indicate selection or preference whereas values lower than 0.5 should be interpreted as avoidance, relative to the other available habitats [56].

**Fig 5:**
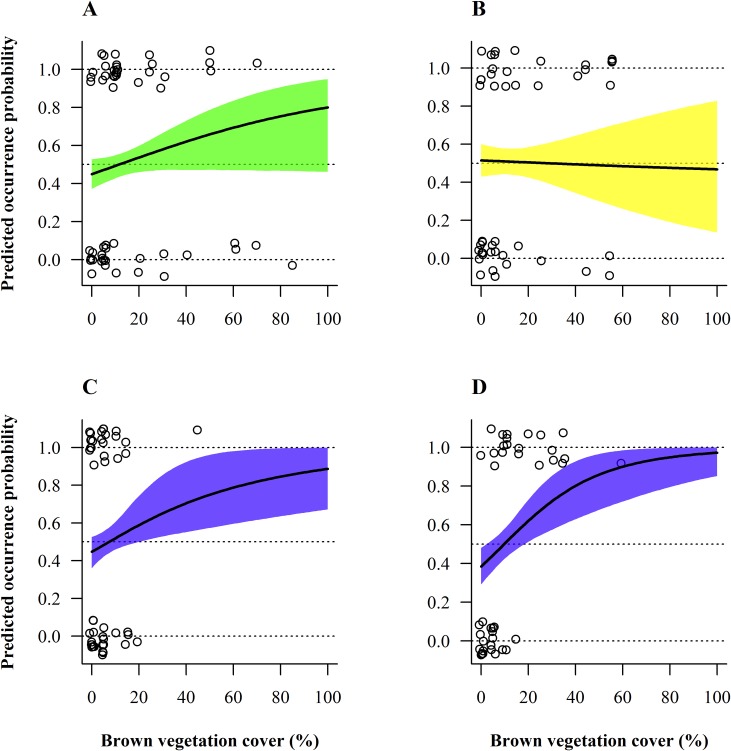
Model-averaged predicted seasonal bird occurrence probability depending on brown ground vegetation cover and month. (A) March. (B) June. (C) September. (D) December. Selection of brown ground vegetation cover by pooled species significantly varied between seasons (months) at the field scale. The four months were selected because they reflect the greatest changes in habitat selection along the annual cycle. 95%-Bayesian credible intervals are depicted by different coloured belts representing contrasted selection patterns. Circles represent raw data of the entire year. Occurrence probabilities greater than 0.5 indicate selection or preference whereas values lower than 0.5 should be interpreted as avoidance, relative to the other available habitats [56].

Overall habitat selection (pooled species) for ground vegetation cover significantly differed across seasons (Tables 2D and 3D). Regarding the green ground vegetation cover, monthly projections for habitat use displayed two distinct selection patterns: in winter (November-March) bird occurrence probability increased linearly with ground vegetation cover, reaching a plateau at around 50%, with parcels covered by < ca 15% green ground vegetation cover being clearly avoided (graphs with blue belts in Fig 4A–4C, 4K and 4L). During spring and until late summer (May-September) the pattern was, in contrast, curvilinear, with birds showing a preference for intermediate green ground vegetation cover with an optimum around 15–60%, peaking at ca 40% (see red belts in Fig 4E–4I). The habitat preference curves shifted gradually and smoothly from one type to the other (see in April and October, i.e. at the two inter-seasons (Fig 4D and 4I). Concerning brown ground vegetation cover, the selection curve significantly changed between summer and winter (Tables 2D and 3D), indicating that brown ground vegetation cover was favoured in autumn and winter (September-February; Tables 2D and 3D and Fig 5C and 5D). In March–August the wide credible intervals precluded interpretation (Fig 5A and 5B).

**Fig 6:**
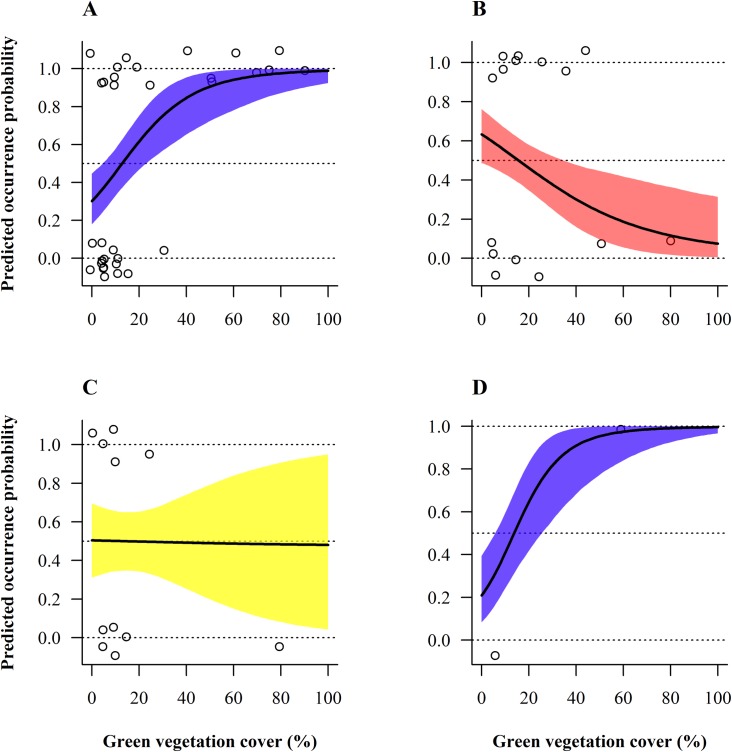
Model-averaged predicted seasonal occurrence probability of thrushes depending on green ground vegetation cover and month. (A) March. (B) June. (C) September. (D) December. Selection of green cover by thrushes significantly varied between seasons (months) at the field scale. The four months were selected because they reflect the greatest changes in habitat selection along the annual cycle. 95%-Bayesian credible intervals are depicted by different coloured belts representing contrasted selection patterns. Circles represent raw data of the entire year. Occurrence probabilities greater than 0.5 indicate selection or preference whereas values lower than 0.5 should be interpreted as avoidance, relative to the other available habitats [56].

**Fig 7:**
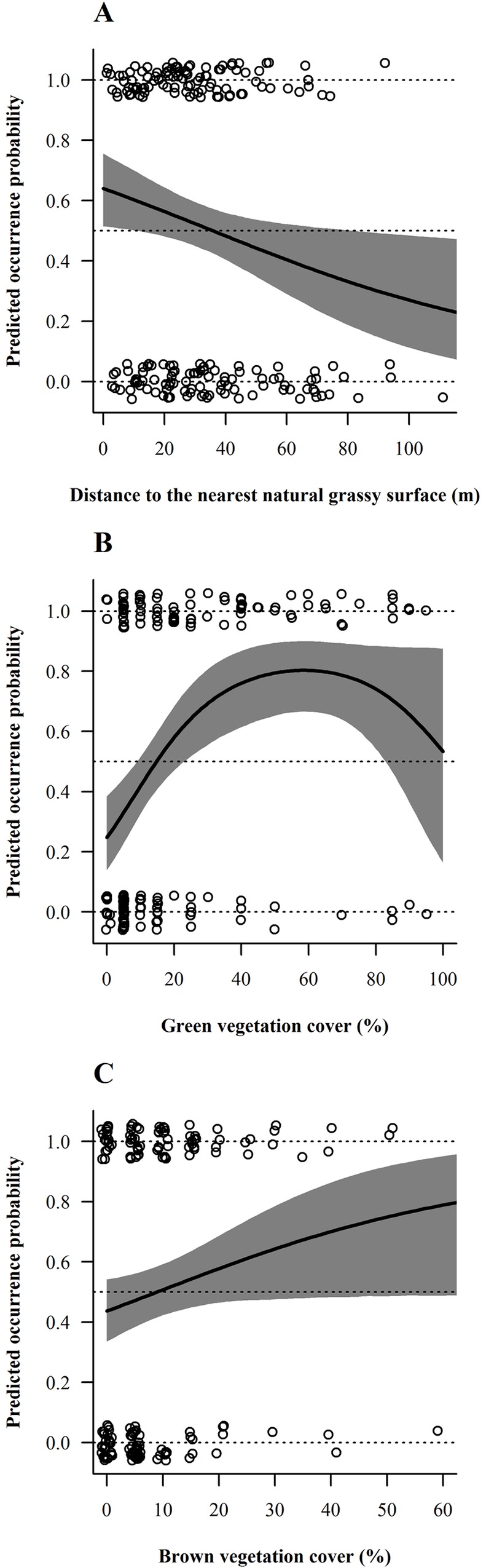
Model-averaged predicted overall occurrence probability of thrushes and finches depending on retained habitat variables. (A) *Turdus* spp. (thrushes) with respect to distance to the nearest natural grassy area. Fringillidae (finches) in relation to (B) green and (C) brown vegetation covers. These habitat selection patterns at the field scale remained constant throughout the year. 95%-Bayesian credible intervals are drawn in grey. Circles represent raw data of the entire year. Occurrence probability was allowed to vary with the habitat variable under consideration, while other explanatory variables present in the average model were held constant at their mean values. Occurrence probabilities greater than 0.5 indicate selection or preference whereas values lower than 0.5 should be interpreted as avoidance, relative to the other available habitats [56].

As for pooled species at the field scale, thrushes favoured parcels where green ground vegetation cover exceeded 50% in winter (November-March; Fig 6A and 6D). In summer (June-July), the opposite selection pattern was observed, with a predicted thrush occurrence probability diminishing with increasing green ground vegetation cover and parcels offering more than ca 15% green cover clearly avoided (Fig 6B). Uncertainty was too high to make reliable predictions for the remaining months (April-May and August-October). Thrushes also tended to select vine parcels located closer to natural grassy areas (< 35 m; Tables 2C and 3C) as depicted by predicted occurrence probability of thrushes decreasing with increasing distance to that type of habitat (Fig 7A), independently of the season.

A clear preference of finches for parcels where green ground vegetation cover amounted to 40–80% could be demonstrated, with maximal occurrence probability at around 60% green ground vegetation cover (Table 2C and Fig 7B). Moreover, finches were more likely to use parcels offering enhanced brown vegetation cover (> ca. 10%; Table 2C and Fig 7C). Both habitat selection patterns were consistent the whole year round (Tables 2D and 3D). As an overview, vegetation structure variables (green and brown ground vegetation cover; Table 1B) played a far more crucial role in explaining bird occurrence compared to vineyard management or distance to landscape variables, which did usually not have any significant effect on birds at the field scale (Table 3C).

## Discussion

Several key findings emerged from this study with respect to the use of vineyards by birds at different spatial scales throughout the year. Overall, bird abundance, species richness and diversity were greater in winter than in summer. At the landscape scale, grove cover (hedges and woody patches) positively influenced bird abundance, species richness and diversity, especially in winter. At the field scale, ground vegetation cover, which undergoes much seasonal variation, appears to constitute the most crucial habitat feature for the avifauna. Finally, bird species-habitat associations also greatly varied according to taxon (thrushes, finches), highlighting the need to consider the distinct resource needs of different guilds if not species, which bears a high relevance for drawing management recommendations for bird-friendly vineyards. While these results originate from only 10 transects and a single region in Switzerland, we are confident that the importance of natural structures and ground vegetation as well as the seasonal variability in habitat selection patterns can be generalized to at least vineyards from other regions [43,50–53] and potentially to other perennial crops such as orchards (e.g. [6,11,71]), if not even to other agroecosystems (e.g. [13,15,24,32,34,72]).

A higher bird abundance, species richness and diversity in our study area in winter can be explained by two non-mutually exclusive arguments. First, south-exposed slopes devoted to viticulture constitute the first locations released from snow in winter, rendering the soil surface of vine plantations among the most readily accessible foraging sites for birds in adverse, snowy conditions. As noted by Laiolo [50], woodland birds such as thrushes, chaffinches or great tits *Parus major* often look then for weed seeds on the ground and invertebrates in the bark of the vines. Alpine chough *Pyrrhocorax graculus*, that make daily movements from the Alpine cliffs to lower elevations in winter, are also attracted by these food sources, exploiting left-over grapes and seeds. Secondly, in winter, many bird species aggregate into larger, intra- and interspecific flocks that move in search for suitable foraging patches [32]. This social behaviour leads to more numerous and richer bird communities than during the breeding season when birds are territorial.

Despite the fact that our landscape scale analysis relies on only 10 transects while groves were scarce in the matrix (0–11%), these woody structures positively influenced overall bird and thrush abundances, species richness and diversity especially during the winter season. The general preference for grove corroborates earlier findings in a variety of agroecosystems that hedges provide foraging, nesting and sheltering opportunities, as well as song posts for the avifauna [1,71–74]. The stronger positive effect of grove in winter might be related to the dominance of forest species which forage in open habitat but rely on woody patches offering sheltering opportunities. Hedges are furthermore known to promote some rare and/or specialist bird species that typically occur among vineyards, such as the cirl bunting *Emberiza cirlus* [75], the rock bunting *Emberiza cia* [76] or the red-backed shrike [16,23,73]. However, they are detrimental to open-land species such as the yellow wagtail *Motacilla flava* [13] and the woodlark, which need large open, “homogeneous” habitats void of woody elements [52,77]. Hedgerow structure and composition is also known to affect birds in a species-specific way [74,78], which might explain why we couldn’t detect any effect of grove presence upon finches at a larger scale. There are pronounced interspecific divergences in the ecological requirements of Fringillidae [79] but our dataset was too coarse to model single species responses.

At the field scale, an overall seasonal shift in vineyards use by birds was observed with respect to green and brown ground vegetation cover. Vineyards with a green ground vegetation cover of 40–100% were visited more often in winter whereas from spring to late summer birds tended to select parcels with an optimum green ground vegetation cover around 40%, while they avoided 100%-vegetated parcels. The preference for brown ground vegetation cover increased in autumn and winter. The strong winter selection for ground vegetated vineyards is certainly driven by the stock of seeds that are picked up by granivorous birds directly from the weed plants [5,7,8], a preference also detected for overwintering birds in orchards [6]. Conversely, the spring-summer preference for soils that are only partly covered by vegetation would result from a diet based primarily on ground-dwelling invertebrates (note that a protein-rich food is essential also for chick growth of granivorous bird species) [80]. Those ground-dwelling invertebrates are hunted by birds walking among vine rows, which is greatly facilitated by the presence of patches of bare ground [37]. In this respect, prey accessibility might be as important as prey abundance for determining food availability in terrestrially foraging insectivorous birds. Thus, prey availability during the breeding season would result from a trade-off between invertebrate abundance and accessibility for this category of birds [24,34–38].

Different vegetation structures are selected by ground-foraging bird species according to their foraging strategy [35]. For instance, thrushes feed principally upon underground-dwelling invertebrates, notably earthworms, although they complete their diet with berries in winter [81]. Our projections for thrushes indicate they would avoid vineyards harbouring more than 15% green ground vegetation cover in summer. This is in line with both their basic trophic requirements and the above abundance-accessibility trade-off that drives food resources availability in the warm season in vineyards. The situation is different in winter when 40–100% ground-greened vineyards are preferred by thrush species, probably because vegetation cover determines to a large extent belowground invertebrate communities which might represent a crucial food substitute to berries in late winter. The selection of parcels located next to natural grassy areas might be explained similarly.

For finches, preferred vineyards had ca 60% green ground vegetation cover. Five out of the six species of finches identified in this study are granivores foraging either directly from herbaceous plants or on the ground all over the year; the chaffinch, the sixth species, feeds mainly upon invertebrates in spring-summer but is absent from vineyards during the breeding season [32]. As a consequence, preference for greater vegetation cover might merely reflect higher seed supply.

### Management recommendations and conclusion

Based on these results, we first recommend to keep and rehabilitate hedges and patches of woody vegetation beyond the boundaries of the grape production area or between vine parcels, this in order to bolster avian biodiversity at the landscape scale. This is in line with the suggestion by Ceresa et al. [66] that even a slight increase in grove cover will benefit avian biodiversity. Although this measure is likely to be quite effective in intensively managed vineyard regions presently void of woody structures [51,82,83], if the objective is to ensure the provision of a genuine pest control service by insectivorous birds, then more heterogeneous farmed matrices would be needed [20,84]. When rehabilitating woody structures, a diverse species composition must be envisioned to provide a broader palette of resources [77].

Another important beneficial measure, to be implemented within the vineyards themselves, is to allow ground vegetation to grow on roughly half of the ground surface, for instance within every second vine row or inter-row. This would represent the best option to sustain bird communities and populations the year-round. Whenever possible, another ideal option would of course be to remove grass mechanically instead of chemically from the «bare ground» row or inter-row, as requested by the organic and biodynamic cultivation protocols. This is because of the potential deleterious effects of herbicide application on plant and invertebrate communities [41, 78], with cascading effects upon avifauna [53], as illustrated for birds in orchards [85,86]. Yet, if half of the Valais vineyards would be half-covered by ground vegetation this would already represent an enormous change for biodiversity (currently 95% of the vineyards in the study area have a total mineral appearance due to extensive herbicide treatment [37]).

Boosting biodiversity among vineyards will probably in the future represent an important asset of grape and wine production, notably in terms of ecosystem functioning and service provisioning (e.g. pest control, combatting soil erosion, enhancing soil fertility, etc.) [1,87]. Thus, in addition to its intrinsic value for human recreation, the promotion of woody structures and grassy ground cover within and adjacent to vineyard parcels paves the way towards a more sustainable grape and wine industry. Similar issues and solutions might be valid for other agroecosystems, especially for other perennial crops such as orchards [6,71,85,86].

This study confirms that multiple species and scales must be considered when dealing with the sheer complexity inherent to the conservation and restoration of farmland biodiversity. It also shows that the land sparing and land sharing concepts, rather than being mutually exclusive options, would greatly benefit from being applied as dual, complementary management schemes [88].

## Supporting Information

S1 Fig Geographic distribution and sampling randomization of study sites. The three shape-coded vs. five letter-coded regional zones regroup transects for random selection of visit order during the breeding and the non-breeding seasons, respectively. Reprinted from Swisstopo under a CC BY license, with permission from Alexandra Frank (see S1 File).
(PDF)

S1 Table Transect characteristics. (PDF)

S2 Table Species list and sample sizes. (PDF)

S1 File Granted copyright permission from Swisstopo. Email-response to copyright request for satellite picture in Fig 1 and maps in S1 Fig (Creative Commons Attribution License (CCAL) CC BY 4.0).
(PDF)
