# Supplementary figures and images for: Temporal and Spatial Scales Matter: Circannual Habitat Selection by Bird Communities in Vineyards
Source: PLoS One. 2017 Feb 1;12(2):e0170176. doi: 10.1371/journal.pone.0170176 (PMC5287466; doi:10.1371/journal.pone.0170176)

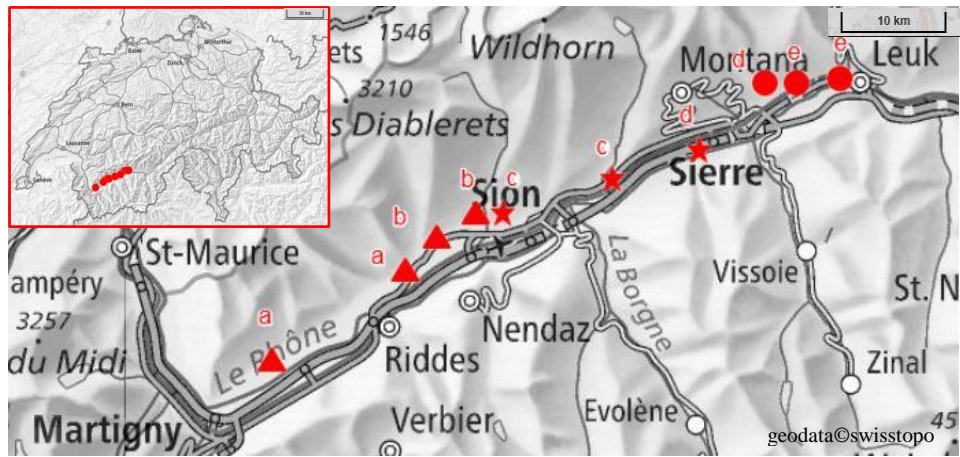

Supplement: S1 Fig — The three shape-coded vs. five letter-coded regional zones regroup transects for random selection of visit order during the breeding and the non-breeding seasons, respectively. Reprinted from Swisstopo under a CC BY license, with permission from Alexandra Frank (see S1 File). (PDF) [file pone.0170176.s001.pdf]
