# Supplementary material for: Temporal and Spatial Scales Matter: Circannual Habitat Selection by Bird Communities in Vineyards
Source: PLoS One. 2017 Feb 1;12(2):e0170176. doi: 10.1371/journal.pone.0170176 (PMC5287466; doi:10.1371/journal.pone.0170176)
Supplement: S1 Table — (PDF) [file pone.0170176.s002.pdf]

Commune (locality)	Altitude (m)	Transect line length (km)	Natural grassy surfaces area (m^2)	Grove area (m^2)	Nr. of Buildings	Nr. of Isolated Bushes & Trees	East coordinate (WGS84)	West coordinate (WGS84)
Fully (Châtaignier)	560	1.11	18510.80	15170.41	19	31	46°09'05.2"N 7°07'33.5"E	46°08'46.7"N 7°06'59.3"E
Chamoson (Gru)	540	1.21	13099.68	407.18	6	14	46°12'21.9"N 7°14'21.6"E	46°11'50.2"N 7°14'15.3"E
Vétroz (Péteille)	600	1.10	13518.74	11830.83	52	38	46°13'36.5"N 7°16'21.6"E	46°13'15.9"N 7°15'42.0"E
Conthey (Sensine)	660	1.17	5739.81	1637.03	27	30	46°14'26.7"N 7°18'15.5"E	46°14'00.3"N 7°17'41.5"E
Savièse (La Soie)	690	0.99	15607.69	11141.88	8	44	46°14'27.8"N 7°19'46.0"E	46°14'14.1"N 7°19'06.4"E
St-Léonard (La Brunière)	610	0.95	13475.23	12034.75	25	35	46°15'32.8"N 7°25'32.5"E	46°15'30.5"N 7°24'49.5"E
Chermignon (Ollon)	560	1.05	7496.66	16735.82	25	44	46°16'39.3"N 7°29'45.2"E	46°16'24.4"N 7°29'02.0"E
Miège (Rotse)	780	1.27	9864.65	15019.69	42	8	46°18'56.0"N 7°33'25.7"E	46°18'51.9"N 7°32'33.2"E
Salquenen (Hell)	650	1.12	8312.61	16986.84	9	23	46°18'53.3"N 7°35'01.7"E	46°19'04.4"N 7°34'19.0"E
Varen (Dude)	700	1.21	52901.93	29425.22	2	91	46°19'06.4"N 7°37'15.2"E	46°18'56.4"N 7°36'28.4"E
**Transect mean ± SE**	635<br>± 75.46	1.12<br>± 0.10	15852.78<br>± 13594.33	13038.97<br>± 8177.62	22<br>± 16	36<br>± 23		
