# Supplementary material for: Temporal and Spatial Scales Matter: Circannual Habitat Selection by Bird Communities in Vineyards
Source: PLoS One. 2017 Feb 1;12(2):e0170176. doi: 10.1371/journal.pone.0170176 (PMC5287466; doi:10.1371/journal.pone.0170176)
Supplement: S2 Table — (PDF) [file pone.0170176.s003.pdf]

**Species - English name**	**Species - Latin name**	**Species recorded at the field scale**	**Total number of individuals at the landscape scale**	**Total number of recorded observations**
Common Buzzard	*Buteo buteo*		17	16
European Honey Buzzard	*Pernis apivorus*		2	1
European Sparrowhawk	*Accipiter nisus*		9	9
Common Kestrel	*Falco tinnunculus*		20	20
Common Wood Pigeon	*Columba palumbus*		34	15
Hoopoe	*Upupa epops*	x	15	11
Black Woodpecker	*Dryocopus martius*		1	1
European Green Woodpecker	*Picus viridis*		4	3
Great Spotted Woodpecker	*Dendrocopos major*	x	12	12
Lesser Spotted Woodpecker	*Dendrocopos minor*		2	2
Eurasian Wryneck	*Jynx torquilla*		2	2
Woodlark	*Lullula arborea*	x	103	71
Eurasian Crag Martin	*Ptyonoprogne rupestris*		5	3
Common House Martin	*Delichon urbicum*		46	6
Meadow Pipit	*Anthus pratensis*	x	24	4
Tree Pipit	*Anthus trivialis*	x	4	3
White Wagtail	*Motacilla alba*	x	43	33
Grey Wagtail	*Motacilla cinerea*	x	16	15
Dunnock	*Prunella modularis*		3	3
European Robin	*Erithacus rubecula*	x	231	228
Common Nightingale	*Luscinia megarhynchos*		1	1
Common Redstart	*Phoenicurus phoenicurus*		3	3
Black Redstart	*Phoenicurus ochruros*	x	526	419
Northern Wheatear	*Oenanthe oenanthe*	x	9	7
Black-eared Wheaear	*Oenanthe hispanica*		1	1
Whinchat	*Saxicola rubetra*	x	3	3
Song Thrush	*Turdus philomelos*	x	6	6
Mistle Thrush	*Turdus viscivorus*	x	239	90
Fielfare	*Turdus pilaris*	x	232	27
Common Blackbird	*Turdus merula*	x	1215	897
Blue Rock Thrush	*Monticola solitarius*		4	3
Garden Warbler	*Sylvia borin*		2	2
Eurasian Blackcap	*Sylvia atricapilla*		89	74
Common Whitethroat	*Sylvia communis*		2	1
Lesser Whitethroat	*Sylvia curruca*		3	3
Western Bonelli's Warbler	*Phylloscopus bonelli*		2	2
Common Chiffchaff	*Phylloscopus collybita*		4	4
Goldcrest	*Regulus regulus*		1	1
Winter Wren	*Troglodytes troglodytes*		23	21
European Pied Flycatcher	*Ficedula hypoleuca*		1	1
Great Tit	*Parus major*	x	433	280
Coal Tit	*Periparus ater*		1	1
Blue Tit	*Cyanistes caeruleus*		85	69
European Crested Tit	*Lophophanes cristatus*		2	2

Marsh Tit	*Poecile palustris*		14	9
Long-tailed Bushtit	*Aegithalos caudatus*		19	3
Eurasian Nuthatch	*Sitta europaea*		4	3
Red-backed Shrike	*Lanius collurio*		15	10
Eurasian Magpie	*Pica pica*	x	57	40
Eurasian Jay	*Garrulus glandarius*	x	301	208
Spotted Nutcracker	*Nucifraga caryocatactes*		4	2
Alpine Chough	*Pyrrhocorax graculus*		141	9
Carrion Crow	*Corvus corone*		147	44
Northern Raven	*Corvus corax*		59	26
Common Starling	*Sturnus vulgaris*	x	145	10
House Sparrow	*Passer domesticus*	x	147	61
Eurasian Tree Sparrow	*Passer montanus*	x	273	94
Common Chaffinch	*Fringilla coelebs*	x	869	270
Common Linnet	*Carduelis cannabina*	x	546	155
European Golldfinch	*Carduelis carduelis*	x	196	86
European Greenfinch	*Carduelis chloris*	x	384	143
Citril Finch	*Serinus citrinella*	x	73	9
European Serin	*Serinus serinus*	x	402	203
Eurasian Bullfinch	*Pyrrhula pyrrhula*		1	1
Cirl Bunting	*Emberiza cirlus*	x	38	34
Rock Bunting	*Emberiza cia*	x	493	300
Undetermined passerines	-		909	323
Undetermined raptors	-		2	2
