# Supplementary material for: Temporal and Spatial Scales Matter: Circannual Habitat Selection by Bird Communities in Vineyards
Source: PLoS One. 2017 Feb 1;12(2):e0170176. doi: 10.1371/journal.pone.0170176 (PMC5287466; doi:10.1371/journal.pone.0170176)
Supplement: S1 File — Email-response to copyright request for satellite picture in Fig 1 and maps in S1 Fig (Creative Commons Attribution License (CCAL) CC BY 4.0). (PDF) [file pone.0170176.s004.pdf]

Dear Mrs. Guyot,

Thank you very much for your request and your interest in our national maps. We are pleased to grant you permission to reproduce and publish the desired map section and the swissimage under a CC BY license.

Purpose

Publication of the manuscript "Temporal and spatial scales matter: circannual habitat selection by bird communities in vineyards" in the open-access journal PLOS ONE under the Creative Commons Attribution License (CCAL) CC BY 4.0 (http://creativecommons.org/licenses/by/4.0/)

Copyright

© 2016 swisstopo (BA16090)

License fee

Considering the purpose of use no license fee will be charged.

Reproduction conditions

1. The copyright is to be cited as source in the visualization.

2. All rights remain vested the Swiss Confederation, represented by swisstopo.

3. The permission is valid for the above mentioned purpose only.

Best regards,
Alexandra Frank

Alexandra Frank
Collaborator Licences

Federal Department of Defence, Civil Protection and Sport
Federal Office of Topography swisstopo
Cartography, Edition / Licences

Seftigenstrasse 264, CH-3084 Wabern

Phone +41 58 469 01 11 reception
Phone +41 58 469 03 42 direct
Fax +41 58 469 04 59
mailto:alexandra.frank@swisstopo.ch
[www.swisstopo.ch]www.swisstopo.ch
